# Sphingolipid Metabolism in Obesity: Bidirectional Regulation and Comparative Perspectives on Plant Sphingolipids

**DOI:** 10.3390/nu18162723

**Published:** 2026-08-20

**Authors:** Mingrui Li, Yunlong Yao, Tianxing Li, Tianqi Cai, Fufangyu Zhao, Yini Fang, Yi Zheng, Chenyu Fei, Can Yang, Wenlong Sun, Mingyan Shao, Lingru Li, Yanfei Zheng

**Affiliations:** 1 Wangqi Academy, National Institute of TCM Constitution and Preventive Medicine, Beijing University of Chinese Medicine, Beijing 100029, China; limingr@bucm.edu.cn (M.L.); 20240931081@bucm.edu.cn (Y.Y.); bucmltx@163.com (T.L.); zhaofufangyu@bucm.edu.cn (F.Z.); fynchn@163.com (Y.F.); 20230931067@bucm.edu.cn (C.F.); yangcan133213@163.com (C.Y.); 2 School of Traditional Chinese Medicine, Beijing University of Chinese Medicine, Beijing 100029, China; 3 Institute of Biomedical Research, School of Life Sciences and Medicine, Shandong University of Technology, Zibo 255000, China; wind0096@163.com (T.C.); wenlongsun0814@163.com (W.S.); 4 Innovation Institute of Integrated Traditional Chinese and Western Medicine, Shandong First Medical University & Shandong Academy of Medical Sciences, Jinan 250117, China; zhengyi@sdfmu.edu.cn

**Keywords:** plant sphingolipids, plant sphinganines, sphingolipid metabolism, obesity

## Abstract

Sphingolipids, a class of lipids, are widely present in the cell membranes of all eukaryotic and some prokaryotic organisms. These lipids play crucial roles in the formation of the lipid bilayer and are categorised into several groups, including sphingomyelins, ceramides, sphingosine-1-phosphate, and glycosphingolipids. In addition to their structural significance, sphingolipids also show bioactivity, regulating various signalling pathways involved in cell growth, differentiation, ageing, and apoptosis, and are closely associated with several chronic diseases, including obesity and type 2 diabetes mellitus. Notably, dysregulated sphingolipid metabolism contributes to obesity-related metabolic dysfunction through altered lipid accumulation, insulin signalling, organelle stress, and inflammation. This review summarises the various mechanisms and recent research advances related to sphingolipids and their association with obesity. It further examines how the metabolic effects of ceramides vary according to their acyl-chain length, enzyme of origin, tissue distribution, and metabolic context, as well as how adipose depot, sex, age, and metabolic phenotype influence the sphingolipid response to obesity. It further considers plant-derived sphingolipids as a compositionally distinct exogenous input to host sphingolipid pools, comparing their structural and enzymatic features with those of mammalian sphingolipids. Pharmacological, dietary, and lifestyle approaches that modify sphingolipid metabolism are also considered. Accordingly, the review summarises our current knowledge regarding the dietary occurrence, intestinal handling, and metabolic effects of sphingolipids and identifies the evidence gaps that currently limit conclusions regarding their relevance to obesity.

## 1. Introduction

Obesity, characterised by the excessive accumulation and abnormal distribution of body fat, has emerged as a major public health challenge worldwide [1]. Obesity is a major risk factor for a range of metabolic complications, including insulin resistance, diabetes, hyperlipidaemia, cardiovascular diseases, and certain types of cancer, thereby posing a serious threat to human health and quality of life [1,2]. With the rapid development of metabolomics and lipidomics technologies, studies have increasingly confirmed that bioactive lipids play key regulatory roles in the development and progression of obesity and its complications [3]. Notably, dysregulated sphingolipid metabolism has gradually become a major focus of research in this field [4].

Sphingolipids, a family of compounds that are ubiquitous in animals, plants, fungi, and some bacteria, act as essential constituents of cell membranes [4]. Moreover, they also participate in cell proliferation, apoptosis, and inflammatory signalling [4,5]. Under normal physiological conditions, sphingolipid metabolism remains in a dynamic state of balance. However, the disruption of this balance can directly impair lipid homeostasis in the body and subsequently contribute to the development of metabolic disorders [6,7].

Obesity is a typical manifestation of abnormal lipid metabolism and is closely associated with dysregulated sphingolipid metabolism [3]. Clinical studies have shown that, in non-alcoholic fatty liver disease, elevations in both the hepatic fat content and the incidence of non-alcoholic steatohepatitis are associated with the production of metabolically harmful, ceramide-rich hepatic liposomes [8]. In addition, extensive metabolomics and lipidomics data suggest that serum and tissue ceramide levels can significantly influence the development of insulin resistance and the prognosis of cardiovascular disease [9,10], suggesting that abnormal sphingolipid metabolism is an important intermediate mechanism linking obesity to metabolic complications. Furthermore, several animal studies have demonstrated that inhibiting ceramide synthesis through inhibition of serine palmitoyltransferase (SPT), the rate-limiting enzyme in the de novo ceramide synthesis pathway, ameliorates insulin resistance and hepatic steatosis and may provide protection against cardiovascular complications [11].

Although the link between obesity and sphingolipid metabolism is well recognised, the underlying molecular mechanisms, the specific functions of different sphingolipids, and the most effective therapeutic targets remain unclear. This review focuses on the relationship between obesity and sphingolipid metabolism and systematically explores the roles and mechanisms of sphingolipid dysregulation in the development of obesity, aiming to provide a theoretical basis for disease prevention and treatment.

## 2. Methods

This narrative review was conducted to synthesise the current evidence on the structure and metabolism of sphingolipids, their bidirectional relationship with obesity, and the comparative features of plant-derived sphingolipids. The review aimed to integrate structural, mechanistic, preclinical, and clinical findings relevant to sphingolipid metabolism as a modifiable node in obesity and its metabolic complications, and the relevant literature was selected accordingly.

Articles were identified through targeted searches of PubMed and Web of Science, covering publications from database inception to 2026. In addition, the reference lists of relevant reviews and original studies were also searched. The search strategy focused on combinations of terms related to sphingolipid structure and metabolism, including “sphingolipid”, “ceramide”, “sphingomyelin”, “sphingosine”, “long-chain base”, “sphingosine-1-phosphate”, “ceramide-1-phosphate”, “glucosylceramide”, “glycosphingolipid”, “serine palmitoyltransferase”, “ceramide synthase”, “sphingomyelinase”, and “sphingosine kinase”, together with terms related to obesity and its associated metabolic phenotypes, including “obesity”, “high-fat diet”, “adipose tissue”, “insulin resistance”, “type 2 diabetes mellitus”, “hepatic steatosis”, “non-alcoholic fatty liver disease”, “endoplasmic reticulum stress”, “mitochondrial dysfunction”, and “lipotoxicity”. A separate set of searches was conducted for plant-derived sphingolipids using the terms “plant sphingolipid”, “phytosphingosine”, “phytoceramide”, “hydroxyceramide”, “glycosylinositol phosphorylceramide”, and “dietary sphingolipid”, both alone and in combination with the obesity-related terms, to ensure that the literature on plant sphingolipids was represented independently rather than only as it appeared within animal-focused studies. Terms related to microbial sphingolipid sources, including “gut microbiota”, “*Bacteroides*”, and “bacterial sphingolipid”, were also searched to capture exogenous contributions to host sphingolipid pools. No date restriction was applied during the search process, allowing seminal studies describing sphingolipid structure and biosynthetic pathways to be retained alongside more recent works.

Priority was given to peer-reviewed articles published in English, including original mechanistic studies, lipidomic and metabolomic analyses, animal intervention studies, clinical and observational studies, and authoritative reviews. Studies were selected according to their relevance to the conceptual framework of this review, with particular emphasis on evidence linking specific sphingolipid species or chain lengths to defined metabolic phenotypes, the enzymatic regulation of sphingolipid flux, and functional comparisons between animal- and plant-derived sphingolipids. Conference abstracts, editorials, and reports in which sphingolipids were measured without mechanistic or phenotypic interpretation were excluded. Where multiple studies addressed the same mechanism, the most recent and methodologically informative work was prioritised, while earlier seminal studies were retained to provide conceptual context. No formal meta-analysis was performed because of the heterogeneity across species, tissues, sphingolipid species examined, analytical platforms, and study designs. The findings were therefore organised narratively into thematic sections covering sphingolipid structure, sphingolipid metabolic pathways, the impact of obesity on sphingolipid metabolism, the impact of sphingolipid metabolism on obesity, and the comparative biology of plant sphingolipids. Throughout this review, the strength of each reported relationship has been indicated by the model system in which it was established. Unless a human study is explicitly cited, statements should be interpreted as being based on preclinical evidence. Notably, the available human data are, with few exceptions, derived from cross-sectional associations rather than interventional studies. The same convention applies to the tables and figure legends.

## 3. Structure of Sphingolipids

Structurally, sphingolipids contain three basic components: a long-chain base (LCB), a fatty acid, and a polar head group. This section describes the structural features shared by sphingolipids and highlights the sphingolipids specific to plants.

The LCB, commonly referred to as sphinganine, is a fundamental component of sphingolipids and serves as the basic structural framework for various sphingolipid species. It is also considered the simplest type of sphingolipid. In plants, LCBs mainly exist in the d18:0, t18:0, d18:1, and t18:1 forms and are linked to C16–C26 fatty acid chains, forming the ceramide backbone [12]. Sphinganines can be classified into three structural classes: unsaturated sphinganines carrying double bonds, saturated dihydrosphinganines, and plant sphinganines containing three hydroxyl groups. Mammals use the first two classes of sphinganines as sphingoid bases, whereas yeast and plants predominantly use plant sphinganines. Plant LCBs may also contain an additional double bond at either the C4 or C8 position [4]. Sphingolipids are formed when sphinganine molecules are acylated with long-chain fatty acids at the 2-amino position and condensed with polar groups, such as sugars or phosphates, at the 1-hydroxyl position. Variations in the length of the LCB, degree of hydroxylation, number and position of double bonds, and the nature of the polar head groups generate a wide range of sphingolipid species [4,13].

**Figure 1:**
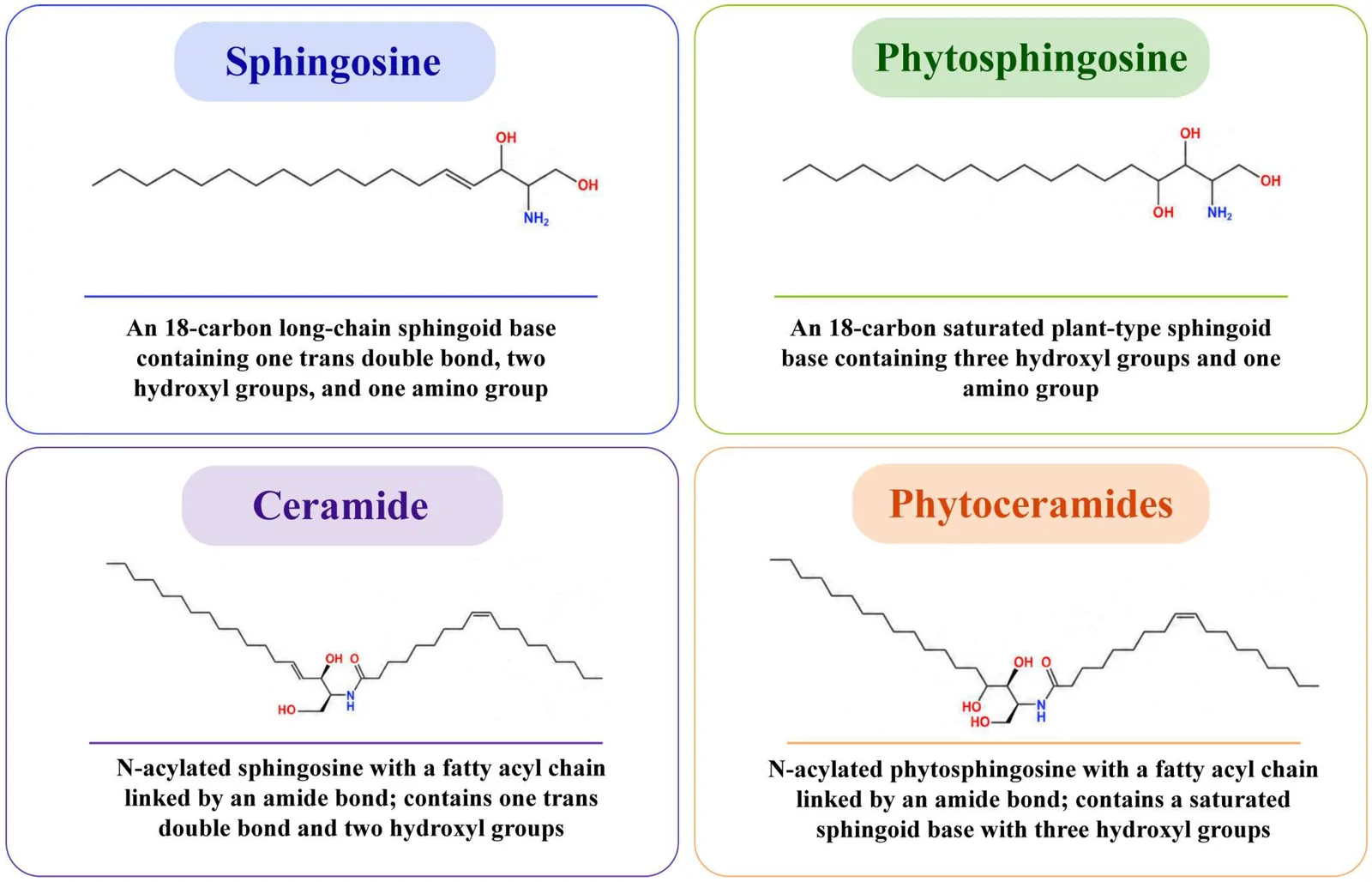
Core structures of mammalian and plant sphingoid bases and their corresponding ceramides. The figure compares sphingosine with phytosphingosine, and ceramide with phytoceramide, illustrating the simplest structural cores from which all complex sphingolipids are assembled. The corresponding ceramides are generated via the amide linkage of a fatty acyl chain to the C2 amino group of each sphingoid base. Only the backbone features that distinguish the two classes are shown. Variations in long-chain base length, the number and position of double bonds, the degree of hydroxylation, and the polar head groups attached at the C1 position are not depicted, and the fatty acyl chains are illustrated in generic form. The structures should therefore be regarded as representative of each class rather than as a comprehensive inventory of the molecular species found in mammalian and plant tissues.

Ceramides are formed when the amino group of the LCB is linked to a fatty acid through an amide bond. Ceramides constitute the core structural framework of sphingolipids, and the attachment of different sugar or phosphate groups at various positions of the ceramide backbone gives rise to a variety of complex sphingolipids [4] (Figure 1). Based on the length of their fatty acid chains, ceramides can be classified as long-chain ceramides, containing C16–C18 fatty acids, or very long-chain ceramides, containing C20–C26 fatty acids. Ceramides of different chain lengths are generated by specific ceramide synthases (CerS1–CerS6) and exhibit distinct biological functions [14]. In plants and some other eukaryotes, ceramide fatty acid chains typically contain 16–28 carbon atoms and are hydroxylated at the C2 position, yielding hydroxylceramides. For example, the addition of a phosphate group to the C1 position of the LCB generates ceramide-1-phosphate (C1P), an important sphingolipid species. Notably, C1P is primarily found in animals but is present only at low levels in plants. Meanwhile, glycosphingolipids are formed through glycosylation at the C1 position of ceramides [4]. These molecules also participate in cell adhesion and signal transduction [15]. Glucosylceramide, formed by the attachment of a glucose moiety at the C1 position of ceramides, is the simplest glycosphingolipid found in living organisms [16]. In contrast, glycosylinositol phosphoceramide (GIPC), which consists of phosphatidylinositol linked to ceramide and modified with hexose or pentose sugars, is the most abundant sphingolipid in plants [17]. Sphingomyelin, another major sphingolipid, is widely distributed in cell membranes, lipoproteins, and other lipid-rich structures. It is formed by the attachment of phosphocholine or phosphoethanolamine to the C1 position of ceramides via the hydroxyl group. In addition to its structural role, sphingomyelin regulates the activity of growth factor receptors and provides binding sites for certain microorganisms and viruses [18].

## 4. Metabolism of Sphingolipids

**Figure 2:**
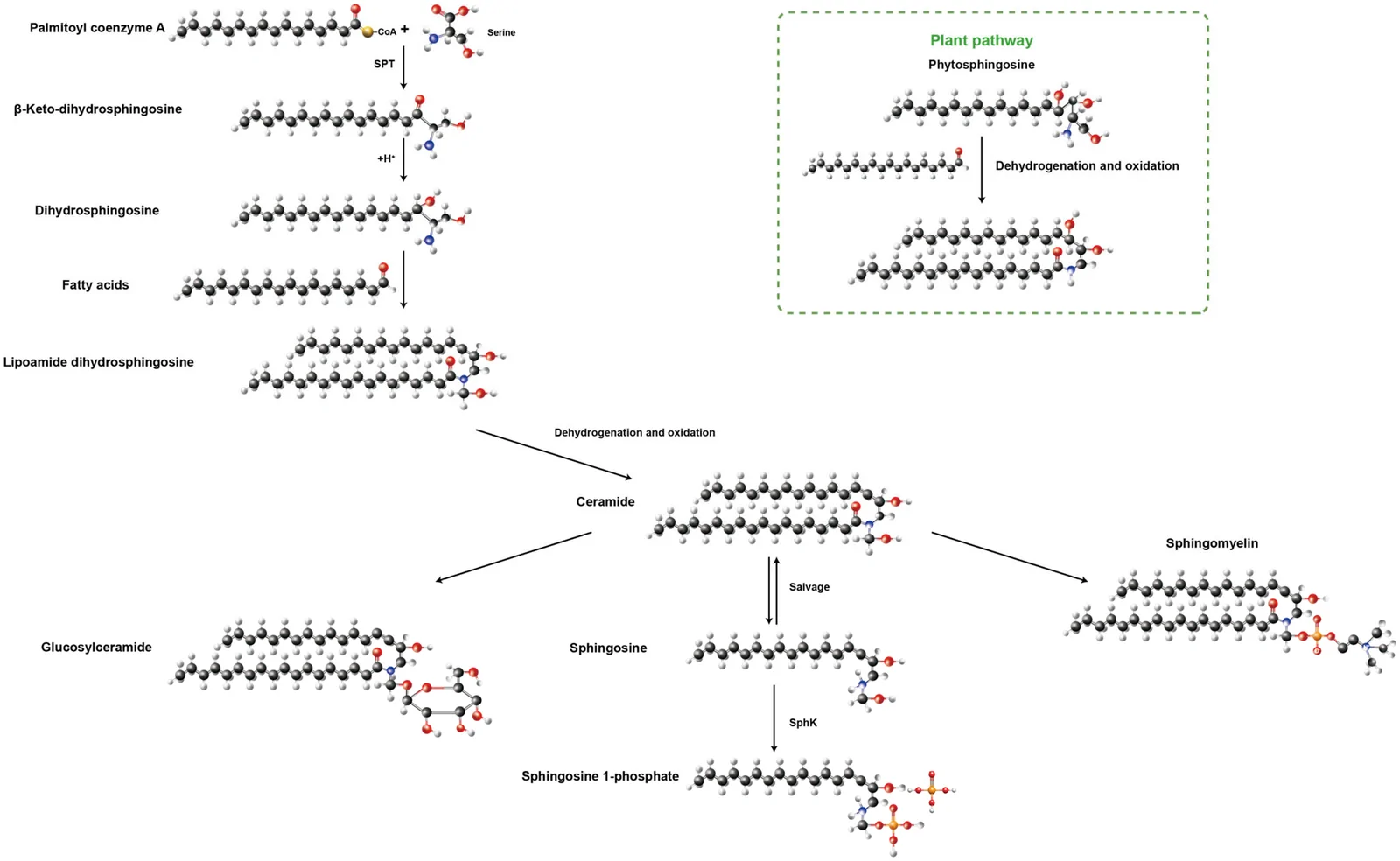
Simplified overview of sphingolipid metabolism. Schematic representation of the three principal routes of ceramide generation—the de novo pathway, the sphingomyelinase pathway, and the salvage pathway—together with the conversion of ceramide into complex sphingolipids and its catabolism to sphingosine and sphingosine-1-phosphate. The schematic is deliberately simplified for clarity: only selected intermediates and enzymes are depicted, single arrows represent several enzymatic steps, and the numerous molecular species with varying acyl-chain lengths, hydroxylation states, and head-group compositions are not differentiated. Subcellular compartmentalisation and inter-organelle ceramide transport are indicated only schematically, and arrow direction does not imply stoichiometry, reversibility, or relative flux. Diet- and gut microbiota-derived sphingolipids are shown as the principal starting points.

The cellular metabolism of sphingolipids starts with dietary intake and gut microbiota-mediated production. In this process, ceramide serves as the central molecule. Sphingolipid metabolism can generally be broken down into three main processes: (i) ceramide generation, (ii) ceramide degradation, and (iii) the synthesis of complex sphingolipids, such as glycosphingolipids and sphingomyelins, through ceramide modification. Ceramide synthesis can be further categorised into three pathways: the de novo synthesis pathway, the sphingomyelinase pathway, and the salvage pathway (Figure 2).

The de novo synthesis pathway is a major route of ceramide synthesis. This route begins with diet-derived substrates, including the amino acid serine and palmitoyl-coenzyme A (palmitoyl-CoA). On the cytosolic side of the endoplasmic reticulum (ER) and associated membranes, serine and palmitoyl-CoA are catalytically converted to 3-ketodihydrosphinganine by serine palmitoyltransferase (SPT), while CoA and CO_2_ are released. Notably, SPT acts as the key rate-limiting enzyme in de novo ceramide synthesis [4]. Thereafter, 3-ketodihydrosphinganine reductase reduces 3-ketodihydrosphinganine to produce dihydrosphinganine, also known as sphinganine [19]. Sphinganine can then enter three pathways. First, it can be phosphorylated by sphinganine kinase to produce sphinganine-1-phosphate, which can subsequently be dephosphorylated back to sphinganine by phosphatases, thereby creating a metabolic cycle. Second, sphinganine hydroxylase can convert sphinganine into 4-hydroxysphinganine, also known as phytosphinganine. Finally, fatty acyl-CoAs of different chain lengths can get attached to sphinganine via the action of ceramide synthases, also known as ceramide *N*-acyltransferases, generating dihydroceramides. This pathway is considered the most important of the three pathways. Subsequently, dihydroceramide desaturase desaturates dihydroceramides to synthesise ceramides [20].

Ceramides participate in four main metabolic pathways. First, they are transported from the ER to the Golgi apparatus, where they are converted into sphingomyelins. These sphingomyelins can then be delivered to lysosomes and hydrolysed back to ceramides by sphingomyelinases, giving rise to the sphingomyelinase pathway [21]. Second, ceramidases can convert ceramides into sphingosine, which can further be phosphorylated by sphingosine kinases to generate sphingosine-1-phosphate (S1P). Third, glucosylceramide synthases can transfer a glucose residue to ceramide, and the resulting glucosylceramides can either be further modified into more complex glycosphingolipids or converted back to ceramide through the salvage pathway. Finally, ceramide kinases can catalyse the formation of C1P [4,22].

Sphingolipids can also be produced by commensal bacteria within the intestinal tract, including *Bacteroides*, *Prevotella*, and *Porphyromonas* species [23]. These bacteria are dominant members of the intestinal microbiota, accounting for approximately 30–40% of the human gut microbiome [24]. Furthermore, they express SPT, the key enzyme required for sphingolipid synthesis, and are capable of producing odd-chain sphinganine bases through the de novo synthesis pathway [4,25]. The sphingolipids produced by these microbial communities can enter the systemic circulation and subsequently be absorbed and processed through various host sphingolipid metabolic pathways [23]. For example, isotopic labelling experiments in germ-free mice have demonstrated that gut bacteria-derived sphingolipids are present in intestinal epithelial tissues and exert biological functions. Hepatic sphingolipid levels were found to be significantly greater in mice colonised with *Bacteroides* strains than in germ-free mice [23]. These findings highlight the importance of the gut microbiota as a significant source of sphingolipids in the body.

Although the sphingolipid metabolism in plants is broadly similar to that in other eukaryotes, some plant-specific features exist [4]. In plants and some fungi, plant sphinganine replaces sphinganine in the downstream biosynthetic steps, although the resulting metabolites and metabolic pathways are otherwise broadly comparable.

## 5. Relationship Between Sphingolipid Metabolism and Obesity

Obesity is frequently associated with abnormalities in sphingolipid metabolism, which, in turn, affect lipid metabolism, leading to insulin resistance, apoptosis, and endoplasmic reticulum stress (ERS). Sphingolipid metabolism and obesity, therefore, exhibit a reciprocal relationship [26].

### 5.1. Impact of Obesity on Sphingolipid Metabolism

**Figure 3:**
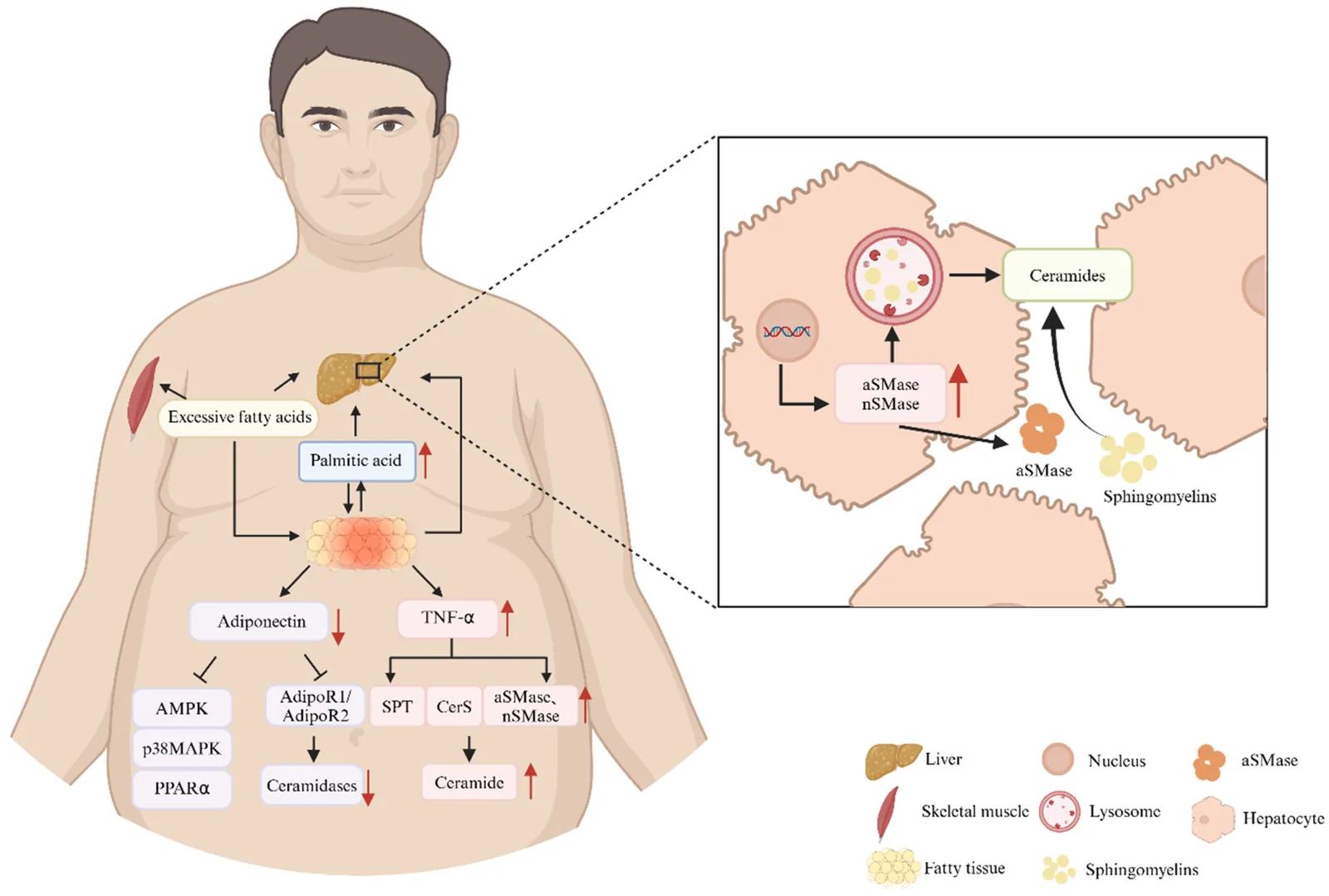
Reported effects of obesity on sphingolipid metabolism. The figure summarises the pathways through which obesity has been reported to alter sphingolipid metabolism in the liver and adipose tissue. Excess fatty acid supply provides substrate for de novo ceramide synthesis via the actions of serine palmitoyltransferase (SPT) and the ceramide synthases (CerS). Pro-inflammatory signalling, represented by tumour necrosis factor-α (TNF-α) acting through p38 MAPK, activates acid and neutral sphingomyelinases (aSMase and nSMase), thereby generating ceramide from sphingomyelin. Adiponectin signalling through adiponectin receptors 1 and 2 (AdipoR1 and AdipoR2), together with AMPK, PPARα, and ceramidase activity in the lysosomal compartment, is shown as a counter-regulatory pathway for ceramide disposal. Arrows indicate only the reported direction of change.

Obesity can influence sphingolipid metabolism through several mechanisms (Figure 3), predominantly increased substrate availability. Adipose tissue releases excess free fatty acids (FFAs), which condense with serine under the catalytic action of SPT to generate 3-ketodihydrosphingosine, thereby increasing sphingolipid biosynthesis. In an acute lipid infusion experiment conducted among healthy women, fatty acid oversupply was found to increase plasma ceramide concentrations and hepatic SPT activity [27], providing the strongest available evidence in humans. In rodents, high-fat feeding was demonstrated to increase ceramide and sphingomyelin content in the liver, adipose tissue, and skeletal muscle [28,29,30], although these studies examined different tissues and sphingolipid species, and none found a uniform increase across all tissues simultaneously. Interestingly, diet-induced changes in intestinal barrier function have been proposed as an additional mechanism of changes in sphingolipid metabolism. Studies have shown that high-fat feeding and genetic obesity increase intestinal permeability and portal endotoxaemia in mice [31,32], while chylomicron formation promotes the intestinal absorption of lipopolysaccharide (LPS) in Caco-2 cells and mice [33]. Although these studies demonstrated that obesity is associated with increased endotoxin exposure, they did not assess adipose sphingolipid synthesis. Consequently, the proposed link between intestinal permeability and adipose sphingolipid metabolism remains inferential. Saturated fatty acids have also been theorised to signal through Toll-like receptor 4 (TLR4). Mice carrying loss-of-function mutations in TLR4 are partially protected against fatty acid-induced insulin resistance [34], and inhibition of ceramide synthesis prevents saturated fat- and glucocorticoid-induced insulin resistance in rodents [35]. However, direct evidence that TLR4 activation upregulates ceramide biosynthetic genes in adipose tissue during obesity is currently lacking, and this mechanism should therefore be regarded as plausible but unproven. The evidence supporting enzymatic regulation, however, is more robust. In mouse models of diet-induced obesity and in primary hepatocytes, hepatic SPT activity and CerS1- and CerS6-dependent ceramide synthesis are both elevated [36]. Similarly, in rat skeletal muscle and cultured myotubes, *SPT2* transcript levels and ceramide content increase following palmitate exposure or high-fat feeding [37]. Notably, these studies do not provide evidence supporting a role for CerS2 or CerS4 in this context. However, other studies show that high-fat feeding preferentially activates CerS6 and increases C16:0 ceramide levels, while whole-body or tissue-specific deletion of CerS6 protects mice against diet-induced weight gain and glucose intolerance [14]. This represents the strongest causal evidence currently available in the field, although it is derived from murine genetic models. In humans, the available evidence remains correlative, with higher *CERS6* expression and C16:0 ceramide content being observed in adipose tissue from individuals with obesity and being positively associated with body mass index [14]. Unfortunately, evidence for sphingomyelinase activation is less consistent. In high-fat-fed and genetically obese mice, adipose tissue and plasma sphingolipid content, together with sphingolipid-metabolising enzymes, including acid and neutral sphingomyelinases, are altered [38,39]. Likewise, hepatic sphingomyelinase activity increases in parallel with ceramide accumulation in rats fed a high-fat diet [40]. Meanwhile, the pharmacological inhibition of acid sphingomyelinase in mice reduces plasma ceramide concentrations and attenuates obesity-associated renal injury [41]. A contribution of both dietary and endogenously synthesised palmitic acid to ceramide synthesis has also been proposed, although this relationship has not yet been examined directly in human adipose tissue [42].

In individuals with obesity, the chronic dysregulation of energy homeostasis arises from a sustained imbalance between energy intake and energy expenditure. Excess triglycerides accumulate in white adipose tissue, and this process is accompanied by adipocyte hypertrophy, hyperplasia, and impaired endocrine function [21]. The expansion of adipose tissue has been associated with tissue hypoxia and macrophage accumulation, both of which are widely considered to contribute to chronic low-grade inflammation [43,44]. Chronic low-grade inflammation has, in turn, been proposed to perturb the enzymes and signalling molecules involved in sphingolipid metabolism. However, the frequently repeated assertion that sphingosine kinase (SphK) activity is suppressed under inflammatory conditions is not supported by direct experimental evidence. Instead, the available data show that SphK1 overexpression in skeletal muscle prevents ceramide accumulation and improves insulin sensitivity in high-fat-fed mice [28]. At present, the direction of SphK regulation in obese adipose tissue therefore remains uncertain.

The expansion of adipose tissue in obesity is accompanied by the altered synthesis and secretion of signalling molecules that may, in turn, influence sphingolipid metabolism. Moreover, the exposure of cultured adipocytes to ceramide, sphingosine, or S1P modifies inflammatory and prothrombotic gene expression [39]. However, the observational findings obtained in genetically obese ob/ob mice and the causal experiments conducted in cultured adipocytes in the same study [39] must be distinguished. Among endocrine alterations, inhibition of ceramide synthesis ameliorated dexamethasone-induced insulin resistance in rodents. The additional claims frequently made regarding glucokinase downregulation and β-cell damage are not supported by the dexamethasone-treated rodent study [35] and are therefore not included here. In contrast to some previous reports, a cross-sectional comparison of individuals with obesity and lean controls found that adiponectin concentrations decreased rather than increased in obesity, whereas leptin concentrations were elevated [45]. Adiponectin acts through adiponectin receptors 1 and 2 (AdipoR1 and AdipoR2), which possess intrinsic ceramidase activity. In mice, adiponectin overproduction or inducible overexpression of AdipoR1/R2 increases ceramidase activity, reduces hepatic ceramide levels, increases S1P concentrations, and improves hepatic insulin sensitivity, whereas receptor deficiency produces the opposite effect [46,47]. The inference that reduced adiponectin concentrations in obesity contribute to ceramide accumulation is therefore mechanistically plausible, although it has not yet been demonstrated directly [47]. Expanded adipose tissue also secretes pro-inflammatory mediators, particularly tumour necrosis factor-α (TNF-α) and interleukin-1β (IL-1β). In primary rat hepatocytes and cells overexpressing the interleukin-1 receptor, IL-1β regulates FoxO1 through a neutral sphingomyelinase 2 (nSMase2)-dependent mechanism [48]. Similarly, in murine macrophages, LPS activates acid and neutral sphingomyelinases and alters ceramide and C1P levels [49]. These findings are indicators of cell-based mechanisms. That TNF-α may upregulate SPT expression and thereby increase ceramide production during obesity in humans remains a hypothesis, and direct evidence from related clinical adipose tissue samples is currently unavailable.

### 5.2. Impact of Sphingolipid Metabolism on Obesity

**Figure 4:**
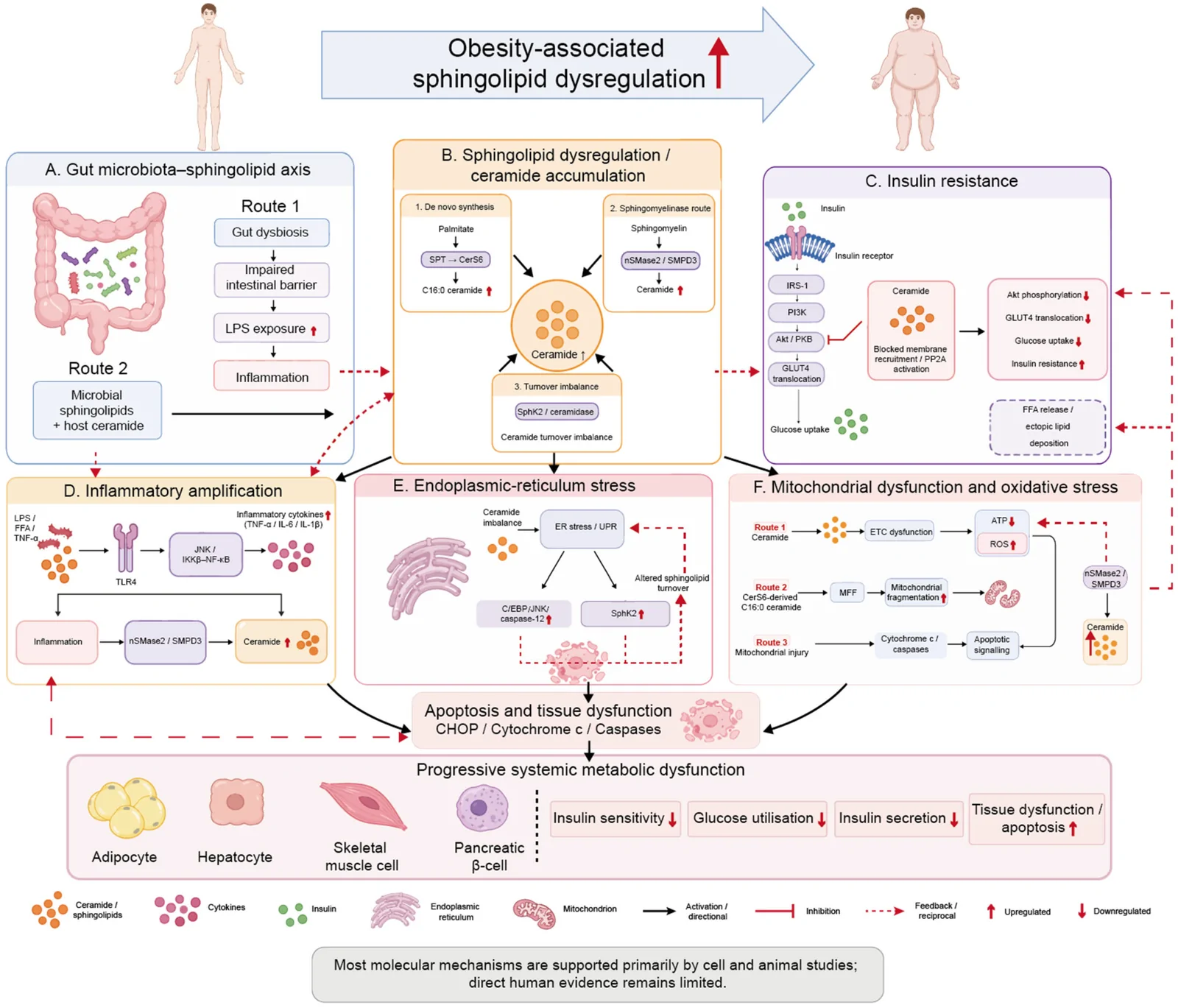
Proposed interactions between altered sphingolipid metabolism and obesity-related metabolic dysfunction. The figure summarises upstream factors proposed to alter the host ceramide pool and downstream mechanisms through which ceramide accumulation may exacerbate obesity-related phenotypes. (**A**) Gut dysbiosis may increase intestinal permeability and LPS exposure, whereas microbiota-derived sphingolipids may either augment or restrain the host ceramide pool depending on the microbial source and metabolic context. (**B**) Ceramide accumulation is represented as arising from enhanced de novo synthesis, sphingomyelin hydrolysis and impaired turnover. (**C**) In the insulin-signalling arm, ceramide limits Akt/PKB activation, GLUT4 translocation and glucose uptake. (**D**) LPS, fatty acids and cytokines act through TLR4–JNK/IKKβ–NF-κB signalling, while inflammatory and oxidative stimuli can promote nSMase2/SMPD3-dependent ceramide generation. (**E**) The endoplasmic reticulum arm is represented by activation of the unfolded protein response and CHOP/JNK-associated injury. (**F**) The mitochondrial and apoptotic arms include impaired electron transport, CerS6-derived C16:0-ceramide/MFF-dependent mitochondrial fragmentation, reactive oxygen species production and apoptotic signalling. The proposed consequences include apoptosis and impaired function of adipocytes, hepatocytes, skeletal-muscle cells and pancreatic β-cells. The mechanisms shown have largely been demonstrated in separate cell and animal models, with limited direct evidence in human obesity, and the scheme should therefore be regarded as a mechanistic framework rather than as a validated biological network. Solid black arrows indicate activation or directionality, red T-shaped lines indicate inhibition, and red dashed arrows indicate feedback or proposed relationships; upward and downward arrows denote upregulation and downregulation, respectively.

Obesity is accompanied by disturbances in sphingolipid metabolism, including the accumulation of ceramides in adipose tissue and other metabolically active organs. Nutrient excess, adipose-tissue dysfunction, gut dysbiosis, inflammation and oxidative stress have each been proposed to alter ceramide production or turnover. Conversely, ceramide accumulation may exacerbate obesity-related metabolic dysfunction by impairing insulin signalling, inducing endoplasmic reticulum stress, promoting mitochondrial injury and facilitating apoptosis (Figure 4). These mechanisms are presented below, together with evidence that sphingolipid dysregulation may reinforce metabolic and inflammatory stress; however, most have been demonstrated in separate tissues or experimental models rather than as a single integrated network [26,50].

First, sphingolipids can suppress components of the insulin-signalling pathway. Evidence for this mechanism is derived almost entirely from experiments in cultured cells, with additional support from a limited number of rodent studies. Protein kinase B (PKB/Akt) is a serine/threonine kinase that is central to the insulin response, regulating hepatic gluconeogenesis and glucose uptake in adipose tissue and skeletal muscle. Following the binding of insulin to its receptors, phosphoinositide 3-kinase (PI3K) converts phosphatidylinositol (4,5)-bisphosphate [PI(4,5)P2] into phosphatidylinositol (3,4,5)-trisphosphate [PI(3,4,5)P3], which recruits PKB to the plasma membrane, where it is activated by PDK1-mediated phosphorylation. Subsequently, activated PKB phosphorylates AS160, relieving its Rab GTPase-activating protein activity and thereby promoting GLUT4 translocation and glucose uptake [51]. Two distinct mechanisms by which ceramides interfere with this signalling cascade have been characterised in insulin-responsive cells. First, ceramide prevents the pleckstrin homology domain of PKB from binding 3-phosphoinositides, thereby preventing PKB recruitment to the plasma membrane. Second, ceramide promotes the protein phosphatase 2A (PP2A)-dependent dephosphorylation of PKB [52]. Consistent with these observations, ceramide, but not sphingomyelin, antagonises insulin signalling and mitochondrial metabolism in C2C12 myotubes [53]. Ceramide has also been reported to inhibit hormone-sensitive lipase and promote CD36 translocation in preclinical models [50]. Evidence at the in vivo level is more limited. In lean mice, administration of a low-density lipoprotein (LDL)-ceramide complex reduces skeletal muscle glucose uptake and increases the expression of pro-inflammatory genes in skeletal muscle. In addition, circulating LDL ceramide contents are higher in individuals with type 2 diabetes, although this represents an observational association rather than evidence of causality [54]. Ceramides have been proposed to act in concert with fatty acids and pro-inflammatory cytokines to activate c-Jun *N*-terminal kinase (JNK), nuclear factor-κB (NF-κB), and Toll-like receptor signalling, thereby contributing to obesity-associated inflammation and insulin resistance [50,55]. This hypothesis is consistent with the partial protection against fatty acid-induced insulin resistance observed in mice carrying loss-of-function mutations in TLR4 [34]. However, this integrative hypothesis is yet to be validated in vivo. The relationship between ceramides and LPS-driven inflammation appears to be bidirectional and has been characterised primarily in cell models. In murine peritoneal macrophages and J774 macrophages, low concentrations of LPS have been found to elicit the maximal release of TNF-α and macrophage inflammatory protein-2, whereas higher concentrations have been shown to be less effective. Evidence shows that ceramide and C1P act as negative regulators of this response [49]. Similarly, in HT-29 intestinal epithelial cells and in mice with dextran sulphate sodium-induced colitis, the inhibition of sphingomyelinase reduces ceramide levels and suppresses LPS-induced interleukin-8 release [56,57]. Nevertheless, these findings have been derived from macrophage and intestinal inflammation models rather than obesity-associated adipose tissue, and their relevance to chronic low-grade inflammation in obesity remains to be established.

Second, sphingolipids can induce ERS. The evidence supporting this mechanism is heterogeneous: the core regulatory relationship between sphingolipid synthesis and the ERS response was previously established in yeast, whereas the metabolic consequences have primarily been demonstrated in mice. The ER is the principal site of protein and lipid synthesis, processing, and transport, and ERS occurs when unfolded or misfolded proteins accumulate [58]. In response, the ER initiates the unfolded protein response (UPR), which increases the synthesis of binding immunoglobulin protein and protein disulphide isomerase while attenuating global protein synthesis. In ob/ob and high-fat-diet-induced obese mice, ERS contributes to insulin resistance and impaired insulin action [59]. These findings indicate that ERS acts as a contributor to obesity-associated metabolic dysfunction in experimental models rather than as a direct cause of obesity. Interestingly, ceramides have been proposed to act as upstream modulators of the UPR [60]. In yeast, the Orm1 and Orm2 proteins negatively regulate SPT. Additionally, their deletion increases sphingolipid synthesis and activates the UPR, an effect that is reversed by SPT inhibition with myriocin [61,62]. Unfortunately, no equivalent rescue experiment in mammals has been reported. In mice, manipulation of *Sgpp2*, which encodes a sphingoid phosphate phosphatase, upregulates characteristic ERS proteins in pancreatic islets, suggesting that the accumulation of sphinganine-1-phosphate may induce ERS in β-cells [63]. However, these findings have been derived from a β-cell model rather than an obesity model. Central nervous system mechanisms linking hypothalamic ceramide accumulation and ERS to energy imbalance and obesity have also been described. In rodents, hypothalamic ceramide accumulation and lipotoxicity reduce sympathetic outflow and brown adipose tissue thermogenesis, favouring fat accumulation [64]. In addition, hypothalamic IKKβ/NF-κB activation links overnutrition to ERS, impaired insulin and leptin signalling, and obesity in mice [65]. Both findings have been derived from murine central nervous system models but have not been confirmed in humans. Notably, the reciprocal regulation of sphingolipid metabolism by the UPR is supported by evidence from mouse liver and hepatocytes, where ERS activates sphingosine kinase 2 (SphK2) and alters the expression of sphingolipid-related genes [66]. Persistent ERS that exceeds the regulatory capacity of the UPR activates C/EBP homologous protein-, JNK-, and caspase-12-mediated apoptosis [67], although this pathway has been characterised in the context of disease pathophysiology other than obesity.

Third, sphingolipids have been proposed to affect lipid metabolism by inducing apoptosis and reducing insulin secretion. This is the least well-supported of the four mechanisms in a metabolic context because most of the underlying experiments were performed in cancer cell lines or isolated organelles rather than in metabolically relevant tissues. Ceramides are established regulators of apoptosis. Ceramide channels formed in the mitochondrial outer membrane increase membrane permeability, permitting the release of pro-apoptotic intermembrane-space proteins such as cytochrome c, which activates caspase-9 and the downstream caspase cascade [68]. This model has been derived largely from biophysical and cell-based systems and has not been tested in vivo in metabolic tissues. Notably, studies show that the inhibition of CerS6 reduces C16-ceramide levels and prevents TNF-α-induced cell death, although this was demonstrated in MCF-7 breast cancer cells rather than in a metabolic model [69]. Ceramides have also been shown to downregulate nutrient transporters, thereby limiting the uptake of fatty acids and amino acids in cultured cells and yeast [70]. With respect to insulin secretion, chronic palmitate exposure induces ERS and apoptosis in MIN6 and other β-cell lines, while adiponectin receptor-associated ceramidase activity protects β-cells and cardiomyocytes from palmitate-induced cell death in mice [46]. Direct evidence that ceramide accumulation impairs preproinsulin expression and insulin secretion in human islet cells is currently lacking. Overall, the proposition that sphingolipid-induced apoptosis contributes materially to the metabolic phenotype of human obesity should be regarded as a mechanistic hypothesis rather than an established mechanism.

Fourth, it has been proposed that ceramides can impair mitochondrial function. Although mitochondrial abnormalities are frequently observed in obesity, the proposed contribution of ceramides to these abnormalities is primarily based on preclinical evidence and cannot currently be regarded as an established mechanism in humans. In isolated rat heart mitochondria, ceramides inhibit electron transfer in complex I and reduce complex III activity, resulting in the increased production of reactive oxygen species [71]. In CerS2-null mice, altered ceramide chain-length composition in liver mitochondria is associated with oxidative stress and reduced complex IV activity [72]. In rats fed a high-fat diet, skeletal-muscle overexpression of carnitine palmitoyltransferase 1 increased mitochondrial fatty acid flux and oxidation, reduced fatty acid esterification into intracellular lipids, and improved insulin sensitivity [73]. These findings implicate the partitioning of fatty acids between mitochondrial oxidation and intracellular lipid storage in the development of high-fat-diet-induced skeletal-muscle insulin resistance; however, the study did not directly assess mitochondrial ceramide accumulation. In contrast, mitochondrial fission represents a well-defined mechanism. Evidence shows that CerS6-derived C16:0 ceramide binds mitochondrial fission factor and promotes mitochondrial fragmentation, mitochondrial dysfunction, and insulin resistance in obese mice and cultured cells [42]. Meanwhile, the ceramide-dependent regulation of mitophagy via the direct binding of the ceramide I35/F52 site to LC3B-II has also been described, providing a plausible mechanism by which ceramides may influence mitochondrial quality control [74,75]. However, both of these studies were performed in cancer models, including FLT3-ITD-positive acute myeloid leukaemia cells, and this mechanism remains to be examined in metabolic tissue.

Another additional route potentially exists. This route lies upstream of the host, in the gut microbiota. The bacterial sphingolipids that reach the intestinal epithelium and liver may not simply just add to the host pool but may also suppress endogenous sphingolipid accumulation. In gnotobiotic mice, *Bacteroides*-derived sphingolipids have been shown to reduce hepatic ceramide levels and improve hepatocyte respiration under conditions of fatty acid overload [76]. However, obesity appears to attenuate this control mechanism. Specifically, a high-fat diet depletes *Turicibacter*, a commensal that produces lipids which lower host ceramide levels and limit weight gain. Notably, the abundance of this genus is reduced in people with obesity [77]. Microbial activity may also regulate sphingolipid metabolism through host enzymes. In mice with nicotine-associated steatohepatitis, intestinal AMPKα stabilises SMPD3 and increases intestinal ceramide levels, whereas the bacterial degradation of luminal nicotine eliminates this stimulus [78]. Evidence from clinical models, albeit associative, remains consistent with these findings. Studies show that plasma sphingolipids partly mediate the association between gut microbiota composition and incident type 2 diabetes [25]. Moreover, washed microbiota transplantation in overweight patients alters the microbiota composition, serum sphingolipid profile, and metabolic indices [79]. However, evidence that convincingly isolates microbial sphingolipids as the mediator of these changes is currently lacking. Furthermore, the relationship is not uniformly protective, since bacterial glycosphingolipids presented by CD1d modulate intestinal invariant natural killer T (iNKT) cell and group 3 innate lymphoid cell (ILC3) responses in opposite directions across different experimental models [80,81]. Moreover, these effects have been defined in the context of intestinal immunity rather than in adipose tissue or the liver.

Sphingolipid metabolism is best viewed as one of several mechanisms that couple nutrient excess, inflammatory stress and gut-derived signals to obesity-related metabolic dysfunction, rather than as a single pathway that integrates all of these processes [21,26,43,44,50,82,83,84,85,86,87,88,89]. Excess fatty-acid supply and chronic low-grade inflammation can disturb sphingolipid synthesis and turnover, favouring ceramide accumulation in metabolically active tissues [14,27,28,29,30,31,32,33,34,35,36,37,38,39,40,41,44,48,49]. Obesity-associated dysbiosis and impaired intestinal barrier function may provide an additional input by altering exposure of the intestine and liver to microbial products and microbiota-derived sphingolipids [23,25,31,32,33,76,77,78,79,80,81]. The direction of this effect appears to depend on the microbial source and metabolic context, because microbiota-derived sphingolipids have been reported either to increase or to restrain the host ceramide pool. Ceramide accumulation has, in turn, been linked to impaired insulin signalling, reduced glucose utilisation and abnormal lipid storage [14,42,50,52,53,54,55,85,86,87]. Insulin resistance may subsequently promote further lipid release and ectopic lipid deposition [50,55,90,91]. Collectively, these findings support reciprocal relationships among lipid oversupply, gut-derived signals and sphingolipid dysregulation, although the evidence has been obtained from different tissues and experimental models.

Organelle stress represents a second proposed mechanism through which sphingolipid imbalance may exacerbate obesity-related metabolic dysfunction. Ceramide accumulation has been associated with endoplasmic reticulum stress, mitochondrial dysfunction, reactive oxygen species production and apoptosis in cell and animal models [42,58,59,60,61,62,63,64,65,66,67,68,71,72,89,92,93,94,95,96,97,98,99]. Within the endoplasmic reticulum, ceramide has been proposed to impair protein and lipid homeostasis, whereas in mitochondria it can disrupt energy production and increase oxidative stress [42,59,60,61,62,63,64,65,66,71,72,94]. Oxidative stress and organelle dysfunction can, in turn, alter sphingolipid-metabolising pathways, providing a potential mechanism through which ceramide accumulation is sustained [66,89,97,98,99,100,101]. Prolonged activation of these processes has been linked to apoptosis or functional impairment in adipocytes, hepatocytes, skeletal-muscle cells and pancreatic β-cells, with consequences for insulin sensitivity and insulin secretion [42,46,53,63,68,69,85,86,87,88,92,102,103].

These mechanisms, together with the upstream routes responsible for ceramide accumulation, the contribution of the gut microbiota to the host ceramide pool, and the membrane platforms at which metabolic, inflammatory, and oxidative signalling converge, differ markedly with regard to the strength of the available evidence. They are therefore summarised according to their level of evidence in Table 1. No interventional human study has yet demonstrated that lowering a specific ceramide species improves metabolic outcomes, and this remains a principal gap between preclinical and clinical studies.

### 5.3. Chain Length, Tissue Distribution, and Metabolic Context as Determinants of Ceramide Action

Based on the mechanisms outlined above, researchers often believe that ceramides are uniformly deleterious lipids. However, the overarching evidence does not support this view. Ceramides have different acyl chain lengths, are produced by different ceramide synthases, and have varying tissue and subcellular distribution profiles. Moreover, the metabolic state in which they accumulate also differs. Each of these variables has been shown to modify, and in some settings reverse, the direction of the observed effect.

Acyl chain length is believed to be the best-documented determinant, with findings from rodent genetic studies and human cohort studies being broadly consistent. Among different ceramides, CerS6-derived C16:0-ceramide has the most firmly established pathogenic role [14]. Very-long-chain ceramide species have been shown to behave differently. Specifically, mice haploinsufficient for CerS2 exhibit reduced hepatic C22–C24 ceramide levels, along with a compensatory increase in C16:0-ceramide levels, impaired β-oxidation, and increased susceptibility to diet-induced steatohepatitis and insulin resistance [104]. This shows that reduced levels of a specific ceramide can worsen metabolic phenotypes. Similar evidence has been obtained from human studies. In the Framingham Heart Study, the plasma C16:0/C24:0 ceramide ratio, rather than total ceramide concentration, was linked to cardiac structure and function [105]. In the same cohort, and in the Study of Health in Pomerania, the balance between very-long-chain and long-chain ceramide species was associated with cardiovascular events and mortality [106]. Furthermore, circulating ceramides predicted cardiovascular outcomes in the population-based FINRISK 2002 cohort [2], while circulating C16:0-ceramide concentrations were found to be increased in patients with heart failure with preserved ejection fraction [107].

Tissue and subcellular distribution are also known to modify ceramide function independently of acyl chain length. In mouse skeletal muscle, the relevant species is CerS1-derived C18:0-ceramide, and the deletion of CerS5 or CerS6 in skeletal muscle does not reproduce the protection conferred by CerS1 deletion [102]. Meanwhile, in the liver, the relevant species is CerS6-derived C16:0-ceramide [42]. Within adipose tissue, the direction of the effect is depot-specific. That is, deletion of *Sptlc2* in thermogenic adipocytes increases energy expenditure and improves glucose homeostasis, whereas the deletion of *Asah1* in the same cells promotes ceramide accumulation and impairs thermogenic function [103]. Consequently, observations from expanding white adipose tissue cannot be directly extrapolated to thermogenic adipose depots.

The suppression of ceramide synthesis is not inherently desirable. Mice lacking CerS3 die during the neonatal period because of skin barrier failure [108], whereas adult mice deficient in CerS2 lose myelin and develop cerebellar degeneration [109]. Individual ceramide species are therefore physiologically indispensable, and the global reduction in ceramides cannot be considered an appropriate therapeutic objective. Therefore, metabolic context is equally important. Within this framework, the reported beneficial effects of individual ceramide species are less anomalous than they may first appear. For instance, increasing hepatic C24:1-ceramide can reduce body weight and improve glucose tolerance in high-fat-fed mice [110], while targeting alkaline ceramidase 3, which increases hepatic C18:1-ceramide levels, can attenuate inflammation and fibrosis in murine models of steatohepatitis [111]. Ceramide accumulation thus appears to be a signal directing nutrient partitioning [70] that becomes deleterious only when nutrient excess is sustained [50,112].

Methodological variations have contributed partly to some of the apparent inconsistency in this literature. Thus, it is more accurate to say that ceramides are context-dependent signals of nutrient oversupply rather than uniformly harmful metabolites (Table 2) [26,50].

### 5.4. Obesity Is Not a Homogeneous Condition: Adipose Depot, Sex, Age, and Metabolic Phenotype as Modifiers of the Sphingolipid Response

The discussion so far has treated obesity largely as a single condition. However, the sphingolipid response to nutrient excess varies according to fat distribution, sex, age, and metabolic phenotype. These sources of heterogeneity must thus be considered while interpreting any general statement relating ceramides to metabolic risk.

Adipose depots are not interchangeable. Paired sampling of human omental and subcutaneous adipose tissue has demonstrated depot-specific lipidomic signatures, including differences in sphingolipid composition, that are more pronounced in individuals with obesity than in lean individuals [113]. Furthermore, the enzymes responsible for ceramide synthesis are distributed unequally. In inflamed intra-abdominal adipose tissue, sphingomyelinase activity, rather than de novo synthesis, accounts for much of the increase in ceramides. *SMPD3* expression is higher in these tissues than in the subcutaneous depot [114]. These depot-specific differences are further influenced by glycaemic status and sex. Among female patients with impaired glucose metabolism having undergone bariatric surgery, several ceramide species, including Cer(d18:1/16:0), Cer(d18:1/18:0), and Cer(d18:1/24:1), were found to be enriched in visceral versus subcutaneous adipose tissue. However, this difference was not observed in men [115]. Notably, studies have shown that reducing adipocyte sphingolipid synthesis promotes subcutaneous adipose tissue browning and an anti-inflammatory macrophage phenotype [116]. In addition, adipocytes differentiated from progenitor cells of visceral origin contain higher levels of ceramides, dihydroceramides, and sphingomyelin than those derived from subcutaneous progenitors, and this pattern is itself influenced by the donor’s metabolic status [117]. Observations made in one adipose depot therefore cannot be generalised to adipose tissue as a whole.

Sex is another important modifier, although its effects are not unidirectional. In a large lipidomic study, most ceramide species were found to show higher concentrations in men than in women around the fifth decade of life, although this difference narrowed or reversed with increasing age [118]. Obese men also exhibited lower insulin sensitivity and higher circulating ceramide concentrations, although differences in skeletal muscle sphingolipid localisation did not account for these differences [119]. Another study found that sex hormone status can also contribute, since plasma Cer(d18:1/24:0) and Cer(d18:1/24:1) concentrations increase after menopause. In women, these levels are inversely correlated with the levels of oestradiol, which reduces ceramide concentrations in vitro [120]. In addition, plasma from postmenopausal women contains higher concentrations of very-long-chain and hydroxylated ceramide species [121]. In mice, testosterone alters the relationship between ceramide levels and insulin resistance, in part via the suppression of CerS6, thereby producing sex-specific hepatic ceramide profiles [122]. Nevertheless, as much of this mechanistic evidence has been derived from animal and in vitro studies, and most rodent experiments have been performed in males, extrapolation to women with obesity remains provisional.

Notably, ageing also modifies the ceramide pool, in some cases independently of adiposity. Longitudinal human lipidomic studies show that the circulating lipidome, including ceramide-containing lipid classes, changes dynamically with age, and that individuals with insulin resistance exhibit an accelerated pattern of change [123]. Targeted analyses further indicate that some species, including Cer(d18:1/18:1) and very-long-chain sphingomyelins, increase with age, whereas others decline [124]. Evidence from younger populations is also emerging in recent years. In children and adolescents, ceramide concentrations were found to be increased under conditions of obesity and were associated with insulin resistance and cardiometabolic risk, whereas sphingomyelins frequently showed an inverse relationship [3]. However, a comparison of adolescents with obesity and adolescents with type 1 diabetes found the highest ceramide and sphingomyelin concentrations in the diabetic population rather than in the obese group, indicating that age, disease type, and metabolic background should be considered together [125]. A mechanistic link with cellular senescence has also been described, with acid ceramidase supporting the survival of senescent cells [126]. However, direct evidence linking adipose tissue ageing to ceramide accumulation in human obesity remains limited.

Individuals with a similar body mass index may also differ substantially with regard to their metabolic phenotype, and sphingolipid burden appears to reflect metabolic phenotype more closely than anthropometric measures. Deep phenotyping studies have shown that individuals with metabolically healthy obesity have lower skeletal muscle ceramide levels than those with metabolically unhealthy obesity despite comparable adiposity, together with a more favourable adipose, hepatic, and systemic metabolic profile [127]. Similarly, earlier studies stratifying overweight and obese individuals according to clamp-measured insulin sensitivity have shown that C18:0-containing skeletal muscle sphingolipids show increased levels in insulin-resistant individuals independently of obesity itself [128], while untargeted serum lipidomics has distinguished metabolically healthy obesity from morbid obesity with type 2 diabetes in women [129]. Prospective cohort studies further support the discriminatory value of individual ceramide species and their ratios. Circulating Cer(d18:1/16:0), Cer(d18:1/18:0), Cer(d18:1/20:0), and Cer(d18:1/22:0) levels have been linked to insulin resistance and incident type 2 diabetes [10,130], while a composite score weighting Cer(d18:1/16:0), Cer(d18:1/18:0), and Cer(d18:1/24:1) against Cer(d18:1/24:0) has been shown to improve the prediction of mortality and cardiovascular events beyond conventional risk factors [131]. However, it must be noted that these outcomes are cardiovascular rather than obesity-specific.

Taken together, the literature shows that adipose depots, sex, age, and metabolic phenotypes all influence the sphingolipid response to nutrient excess, such that no single ceramide signature can be ascribed to obesity as a single condition. The strongest evidence in humans is related to adipose depots and metabolic phenotypes, for which findings from paired-tissue studies, deep phenotyping, and prospective cohorts are broadly consistent. By contrast, evidence regarding sex differences relies more heavily on animal and in vitro studies, while direct evidence linking adipose tissue ageing to ceramide accumulation in human obesity remains limited.

## 6. Plant Sphingolipids as an Exogenous Input to Host Sphingolipid Metabolism

In the preceding sections, sphingolipid metabolism has largely been described as an endogenous process, modulated by dietary fatty acid supply and the gut microbiota. Plants, however, also synthesise sphingolipids that are structurally distinct from their mammalian counterparts. Because plants account for a substantial proportion of the human diet, these sphingolipids are often consumed by humans daily. However, whether they enter the host sphingolipid pool in amounts sufficient to modify it, and whether any such modification has metabolic consequences in obesity, are separate questions. At present, there are different levels of evidence regarding these matters.

### 6.1. Dietary Occurrence and Intestinal Handling

Dietary glucosylceramides of plant and fungal origin are grouped together wherever the evidence relates to properties shared by both classes. Both groups of glucosylceramides are built on long-chain bases that differ from mammalian sphingosine, principally 4-hydroxy-8-sphingenine and 4,8-sphingadiene in plants, and 9-methyl-4,8-sphingadiene in fungi. However, these long-chain bases share a pattern of limited intestinal uptake.

At present, quantitative estimates of dietary intake remain scarce. An analysis of twelve nutritionist-prepared Japanese meals identified cerebrosides, primarily glucosylceramide, and sphingomyelin as the major dietary sphingolipid classes. The total sphingolipid intake was estimated at 292 and 128 mg/day for high-calorie meals and 81 and 45 mg/day for low-calorie meals, respectively, although plant and animal contributions were not distinguished [132]. Glucosylceramide has been detected in crop tissues and crop-processing by-products, with apple pulp showing the highest concentration among the materials examined (0.94 mg/g) [133]. In addition, GIPC was detected in all 13 vegetables examined at concentrations of 3–20 mg/100 g wet weight, corresponding to an estimated dietary intake of approximately 28 mg/day in Japan [134]. However, the gastrointestinal digestion, absorption, and nutritional effects of dietary GIPC in humans remain unknown.

The intestinal fate of ingested plant glucosylceramide has been characterised primarily in rodent models and Caco-2 cells. The rat intestinal mucosa hydrolyses maize glucosylceramide at levels comparable to those observed for mammalian-derived substrates, indicating that hydrolysis is not the limiting step [135]. Instead, uptake appears to be limiting, since the plant-derived long-chain base 4,8-sphingadienine accumulates less efficiently in Caco-2 cells than sphingosine, although verapamil increases its accumulation, implicating P-glycoprotein-mediated efflux in this process [135,136]. This pattern has also been confirmed in thoracic duct-cannulated rats [137]. After oral administration of maize glucosylceramide, a small fraction of its plant-derived long-chain base, 4t,8c-sphingadienine, was recovered from thoracic-duct lymph and detected in the ceramide fraction, although cumulative lymphatic recovery was very low [138]. These findings indicate that dietary plant glucosylceramide is efficiently hydrolysed but poorly absorbed and only partly reincorporated into ceramides; however, no estimate of the contribution of glucosylceramide-derived plant long-chain bases to circulating or tissue ceramide pools is currently available in humans [135,136,137].

### 6.2. Structural Determinants of Divergent Metabolic Handling

The structural features that distinguish plant sphingolipids are not metabolically inert, because several mammalian enzymes discriminate between plant- and mammalian-type substrates. Mammalian sphingosine kinase 1 phosphorylates sphingosine, dihydrosphingosine, and 4,8-sphingadienine, but not 4-hydroxylated bases such as phytosphingosine [139]. Consequently, the conversion that channels sphingosine into sphingosine-1-phosphate signalling is not readily available to the most characteristic plant sphingoid base. Conversely, human alkaline ceramidase preferentially hydrolyses phytoceramide [140]. When phytosphingosine-1-phosphate is generated, its signalling overlaps with, but is not identical to, that of sphingosine-1-phosphate [141]. These observations, derived from non-metabolic cell types, indicate the presence of divergent metabolic handling rather than direct metabolic activity.

### 6.3. Effects on Obesity-Related Metabolic Phenotypes

Direct evidence that plant sphingolipids modify obesity-related metabolic phenotypes is limited, and its interpretation depends on the specific compound being evaluated. In genetically obese Zucker fatty rats, dietary supplementation with animal-derived sphingomyelin and plant-derived glucosylceramide for 45 days was found to reduce the hepatic lipid content, plasma non-high-density lipoprotein (non-HDL) cholesterol levels, and serum insulin concentrations, while only glucosylceramide was found to upregulate adiponectin [142]. This was, however, a genetic rather than a diet-induced model, and body weight and fat mass were not the primary study outcomes. In another study, oral phytosphingosine was reported to improve high-fat-diet-induced glucose intolerance in mice [143]. Evidence showed that phytosphingosine activates GPR120 with a potency comparable to that of α-linolenic acid [144], providing a plausible link to metabolic regulation. Nevertheless, this mechanism remains to be tested in an animal model of obesity.

Clinical evidence from human studies is even more limited and largely comes from studies on phytosphingosine. In the only trial reporting metabolic endpoints, twelve men with metabolic syndrome were treated with phytosphingosine 500 mg twice daily (or placebo) for four weeks per treatment period in a double-blind crossover study. Total cholesterol, LDL cholesterol, and fasting glucose were found to be lower during phytosphingosine treatment, and the intravenous glucose tolerance test K-value increased by 9.9%. However, body weight and fat mass remained unchanged [145].

### 6.4. Interaction with the Gut Microbiota

Because their absorption is limited, a substantial proportion of ingested plant glucosylceramides reach the distal intestine, where they become available to the gut microbiota. Previously, *Blautia glucerasei* was found to produce a glucosylceramide-specific glucosylceramidase that hydrolyses diverse plant glucosylceramides to ceramide while leaving related glycosphingolipids intact [146]. Interestingly, the evidence indicates that plant glucosylceramides may also have prebiotic functions. For instance, dietary koji (*Aspergillus oryzae*) glycosylceramide was found to increase the abundance of *Blautia coccoides* in the faecal microbiota of mice [147]. However, the glucosylceramide source in this study was fungal, with its 9-methyl-branched sphingoid base not being present in plants. Moreover, no metabolic endpoints were assessed. Thus, whether plant glucosylceramides modify bacterial sphingolipid synthesis and thereby influence hepatic ceramide content [23] is yet to be examined.

### 6.5. Mechanistic Parallels, Safety Considerations, and Unresolved Questions

Some regulatory features are conserved between plant and mammalian sphingolipid metabolism. A structural analysis of the *Arabidopsis* SPT-ORM1 complex demonstrates that ceramide binding is required for ORM1-dependent repression of SPT activity [148], a mechanism analogous to Orm-mediated regulation in other eukaryotes [61] that has been established in a plant system. With respect to inflammatory signalling, plant sphingolipids have been shown to inhibit the nuclear translocation of the NF-κB p65 subunit and IκBα degradation induced by LPS or TNF-α in differentiated Caco-2 cells [149]. Given the limited systemic availability described above, such intestinal effects may ultimately prove more relevant than systemic changes.

Short-term tolerability data are reassuring but remain limited. Phytosphingosine (1 g/day for four weeks) produces only minor gastrointestinal adverse effects [145], and konjac glycosylceramide (six weeks of administration) has not been linked to any adverse events [150]. Nevertheless, long-term safety data are unavailable. Fumonisin B1, a mycotoxin produced by *Fusarium* species growing on maize, is a sphingoid base analogue rather than a dietary sphingolipid. It inhibits ceramide synthase, increases the sphinganine/sphingosine ratio, and is associated with renal and hepatic tumours in rodents [151,152]. Although this plant-derived compound poses a contamination risk, its properties are not intrinsic to all plant sphingolipids. Nevertheless, the evidence indicates that sphingoid base analogues found in plant-derived materials warrant individualised scrutiny.

Based on the current evidence, it is prudent to infer that plant sphingolipids are compositionally distinct as dietary inputs. Overall, their contribution to host sphingolipid metabolism under conditions of obesity remains to be established, and their value as dietary interventions for the management of metabolic disease is yet to be clearly elucidated (Table 3). Taken together, dietary, microbial, adipose-derived, and intracellular stress signals may converge on ceramide metabolism and downstream metabolic injury.

## 7. Targeting Sphingolipid Metabolism: Pharmacological Approaches and Translational Status

### 7.1. Direct Pharmacological Modulation of Sphingolipid Synthesis, Turnover, and Signalling Pathways

Inhibition of SPT, the rate-limiting enzyme in the de novo sphingolipid synthesis pathway, is the most thoroughly characterised pharmacological approach. In mice with high-fat-diet-induced obesity, myriocin (0.5 mg/kg; every alternate day for four weeks) was reported to reduce gastrocnemius ceramide levels from 141.5 ± 15.8 to 94.6 ± 10.2 nmol/g dry weight and reverse glucose intolerance and insulin resistance [153]. However, this compound was not developed for metabolic indications because it was originally characterised as an immunosuppressant, with a reported potency 10- to 100-fold greater than that of cyclosporin A in murine lymphocyte assays [154]. Therefore, the global inhibition of the first committed step in sphingolipid synthesis remains only a mechanistic probe at present. In contrast, the isoform-selective inhibition of ceramide synthases is chemically achievable and potentially effective. P053 shows a half-maximal inhibitory concentration of approximately 0.5 μM against CerS1 but is at least one order of magnitude less potent against other isoforms. In high-fat-fed mice, P053 was found to increase fatty acid oxidation in skeletal muscle and limit adiposity, although it failed to prevent diet-induced insulin resistance [155]. Unfortunately, although the causal evidence for the CerS6 isoform in obesity is the strongest [14], no CerS6-selective inhibitor is currently available. However, other studies have shown that the depletion of dihydroceramide desaturase 1 improves insulin resistance and hepatic steatosis in mice. At present, this remains the best-characterised target within the pathway [50], although a recent study in rats attributed the beneficial effects of both myriocin and Des1 antisense knockdown to reduced sn-1,2-diacylglycerol levels in the plasma membrane rather than to reduced hepatic C16:0-ceramide levels alone [156].

Notably, two catabolic pathways have also been explored. Pharmacological inhibition of acid sphingomyelinase was found to lower plasma ceramide and attenuate obesity-associated organ injury in mice [41]. In LDL receptor-deficient mice, amitriptyline could attenuate steatohepatitis and atherosclerosis while lowering glucose, lipids, and insulin resistance [157]. However, this drug has only been approved for the treatment of depression [158] and shows substantial polypharmacology. Hence, its repurposing for metabolic disease remains to be established as a viable therapeutic option. Conversely, the intrinsic ceramidase activity of AdipoR1 and AdipoR2 [46,47] can be enhanced through receptor agonism. In mice, PEGylated (polyethylene glycol-conjugated) AdipoRon derivatives reduce hepatic, pancreatic, and circulating ceramide species and improve insulin sensitivity during high-fat feeding. In addition, AdipoRon normalises the ceramide-to-S1P ratio and reduces albuminuria in db/db mice without altering body weight [159,160]. At present, however, no compound belonging to this class has entered clinical trials.

The two remaining approaches have been tested clinically, albeit not for metabolic disease. In an early trial, the sphingosine kinase inhibitor ABC294640 (500 mg twice daily) transiently reduced plasma S1P concentrations in patients with advanced solid tumours. However, a subsequent phase II trial in metastatic prostate cancer did not meet its primary endpoint [161,162]. The approved S1P receptor modulator fingolimod was found to alter the lipid profile of patients treated for multiple sclerosis, although ceramide concentrations, S1P concentrations, and insulin sensitivity were not measured [163]. Interestingly, the inhibition of glucosylceramide synthase shows the greatest potential for the clinical translation of preclinical data. In one preclinical study, Genz-123346 reduced glucose and glycated haemoglobin concentrations, improved glucose tolerance, and enhanced skeletal muscle insulin signalling in Zucker rats and mice with diet-induced obesity [164]. Meanwhile, eliglustat, which targets the same enzyme, has been approved for the long-term treatment of adults with Gaucher disease type 1 [165]. However, the compound with demonstrated metabolic efficacy and the approved drug are different agents, and no glucosylceramide synthase inhibitor has yet been evaluated for metabolic indications (Table 4).

### 7.2. Diet- and Lifestyle-Induced Modulation of Ceramide Species

Diet, energy balance, and physical activity alter the sphingolipid pool associated with obesity through effects on substrate supply, catabolic signalling, and nutrient partitioning. These interventions are also the most readily applicable in clinical practice. However, the evidence linking defined interventions to changes in specific ceramide species, and subsequently to metabolic outcomes, is uneven (Table 5).

Saturated fatty acid intake is the dietary variable most consistently associated with ceramide accumulation, although the available human evidence has been obtained following acute or short-term exposure rather than habitual dietary intake. Fatty acid oversupply increases circulating ceramide concentrations and hepatic SPT activity in lean volunteers [27]. A randomised overfeeding study showed that saturated and polyunsaturated fat produced comparable weight gain but had different effects on hepatic fat accumulation and ceramide responses [166], with C16:0-ceramide being most consistently implicated. At the level of overall dietary patterns, plasma ceramide concentrations were found to predict incident cardiovascular disease, and in the PREDIMED trial, this association was modified upon the initiation of a Mediterranean diet [167]. In a 12-month randomised parallel trial involving adults at high cardiovascular risk, participants assigned to the Mediterranean-diet group received individual and group dietary-training sessions at baseline and every three months, with adherence assessed using a 14-item questionnaire and personalised dietary advice, whereas the control group received low-fat-diet counselling at the same intervals. Compared with the low-fat-diet group, the Mediterranean-diet group showed lower serum C24:0 and C18:0 ceramide concentrations and a higher C24:0/C16:0 ratio at 12 months [168], thereby shifting the ceramide profile away from the C16:0-weighted pattern associated with cardiometabolic risk. However, these cohorts were selected on the basis of cardiovascular rather than obesity-related endpoints.

The effectiveness of two additional dietary interventions is supported mainly by indirect or preclinical evidence. A controlled intervention with arabinoxylan-oligosaccharides in overweight adults was found to reduce a small number of plasma ceramide and hexosylceramide features within a broader multi-omic response [169]. However, no human study has yet linked a defined fibre intervention to changes in specific ceramide species together with improvements in metabolic outcomes associated with obesity. For *n*-3 fatty acids, the evidence is weaker than the frequency of research might suggest. In patients with fatty liver disease, fenofibrate was found to lower plasma ceramide concentrations more effectively than omega-3 carboxylic acids, which primarily affected lipid mediators rather than ceramide species [170].

**Figure 5:**
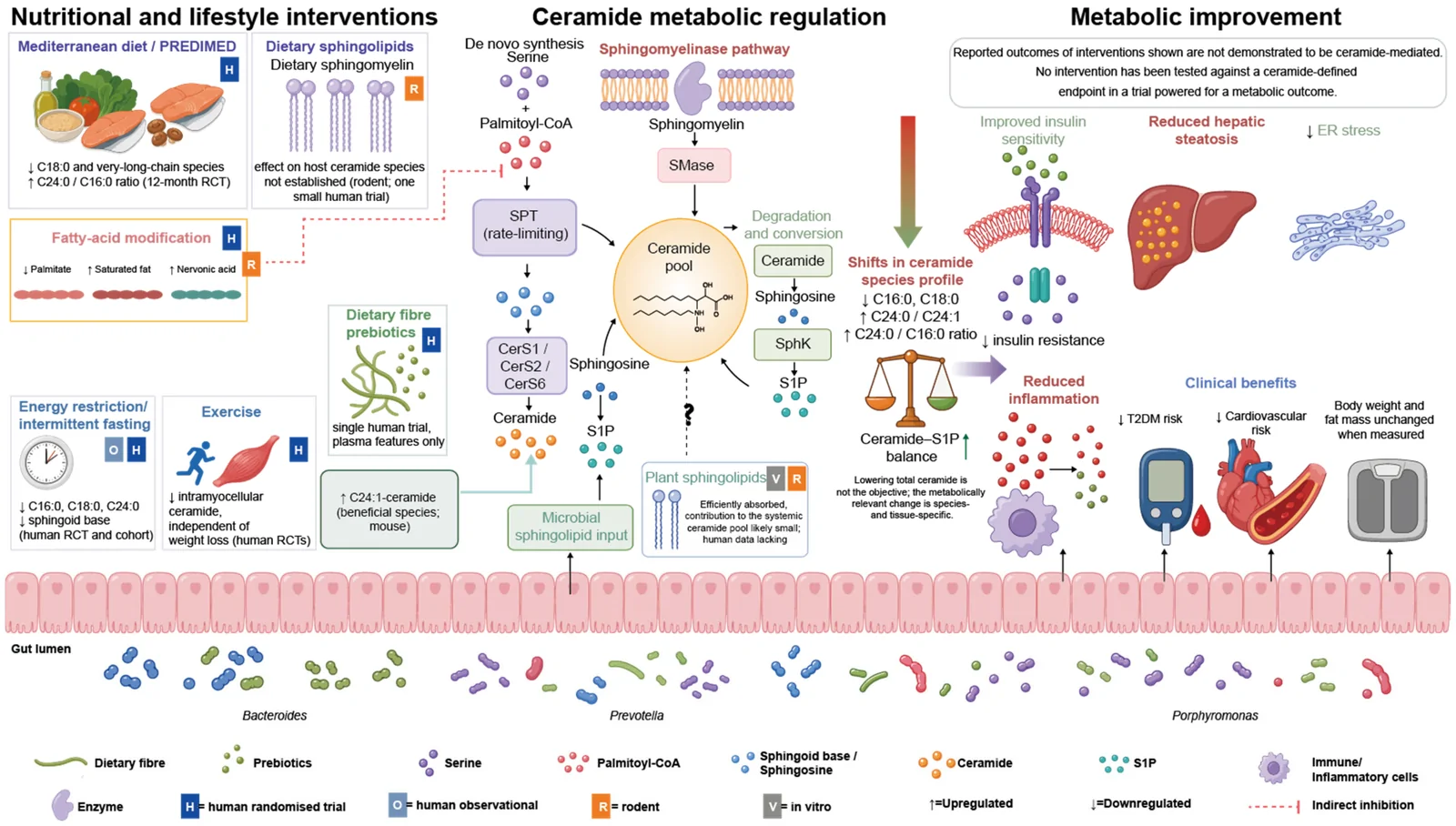
Nutritional and lifestyle interventions reported to modify ceramide metabolism, along with associated metabolic outcomes. The figure is arranged in three columns: the interventions themselves, the steps of ceramide metabolism they target, and their metabolic and clinical outcomes. The effects of Mediterranean-style diets, dietary sphingolipids and sphingomyelin, dietary fibre and prebiotics acting through the gut microbiota, energy restriction and intermittent fasting, exercise, and plant-derived sphingolipids on de novo synthesis via serine palmitoyltransferase (SPT) and ceramide synthases, the sphingomyelinase pathway, ceramidase-dependent degradation, fatty acid modification of the acyl chain, and the ceramide–S1P balance are shown. Font colors are used for visual differentiation only and do not denote the strength of evidence. Solid arrows indicate pathway direction or the direction of the reported change, whereas red dashed inhibitory lines indicate indirect inhibition. Upward and downward arrows denote increases and decreases, respectively. The question mark indicates an unresolved relationship for which the available evidence remains insufficient. H, O, R, and V indicate evidence from human randomised trials, human observational studies, rodent studies, and in vitro studies, respectively.

Energy restriction and exercise have the most consistent supporting evidence in humans. Two years of sustained caloric restriction in non-obese adults can reduce circulating C16:0, C18:0, and C24:0 ceramide concentrations in a manner consistent with improved glucose control through an adiponectin–ceramide axis [171]. Similarly, one month of diurnal intermittent fasting in overweight and obese adults can lower several plasma sphingoid bases and sphingomyelin species [172]. Notably, exercise also decreases intramyocellular ceramide independently of weight loss. Aerobic exercise can reduce skeletal muscle ceramide levels, unlike diet-induced weight loss with comparable metabolic benefits [173]. Furthermore, structured exercise following gastric bypass surgery produces additional reductions in skeletal muscle ceramide species beyond those achieved with surgical weight loss alone [174]. Nevertheless, for several of these interventions, the relevant species- and compartment-specific changes have not yet been demonstrated in human tissues. Collectively, nutritional and lifestyle interventions may modify ceramide metabolism through effects on substrate supply, sphingomyelin turnover, ceramidase-dependent degradation, and the ceramide-S1P balance (Figure 5).

## 8. Limitations of Current Evidence

Several challenges limit the strength of the conclusions that can be drawn from the current literature. Most mechanistic data are derived from rodent models of diet-induced obesity, which differ from human obesity in terms of adipose depot distribution, ceramide chain-length profiles, and the time course of disease development. Moreover, the strongest causal evidence has been derived from tissue-specific rodent models [14,102]. The corresponding human evidence is largely correlative and is based predominantly on circulating sphingolipids [130,167], whereas the tissues of greatest pathophysiological relevance—the liver, skeletal muscle, and pancreatic islets—are sampled much less frequently [128]. Circulating sphingolipid concentrations are therefore an imperfect surrogate for tissue sphingolipid levels. In addition, experimental conditions also limit extrapolation. In vitro studies often employ palmitate concentrations that exceed physiological plasma levels, while rodent studies typically use male animals fed diets containing 45–60% fat for varying durations.

Several of the pathways described—including ORM/ORMDL-dependent feedback regulation of SPT, TLR4- and cytokine-mediated regulation of ceramide biosynthesis, and ceramide-mediated apoptosis and mitochondrial injury—are supported primarily by evidence from yeast, heterologous expression systems, or cultured cells. Thus, their operation in human metabolic tissues remains to be demonstrated [34,61,67,72,175].

Moreover, directionality is also difficult to establish. Circulating ceramide concentrations decrease following caloric restriction, exercise, and bariatric surgery [171,173,176]. However, because these interventions affect adiposity as a whole, a ceramide-specific effect cannot be isolated, and it remains unclear whether ceramide accumulation drives metabolic dysfunction or is a consequence of this process. Comparisons across studies are further complicated by differences in lipidomic platforms, the ceramide species measured, and whether chain-specific or total ceramide concentrations are reported [177,178]. In addition, few studies simultaneously assess enzyme activity, tissue and circulating ceramide species, and metabolic outcomes in the same individuals. Consequently, concurrent changes across compartments cannot, by themselves, establish mediation.

Evidence is even more limited for the exogenous and therapeutic aspects of the field. For plant sphingolipids, the available human evidence relates primarily to phytosphingosine of non-plant origin rather than plant glucosylceramide [145]. No clinical trial has evaluated adiposity, glycaemia, or liver enzymes as primary endpoints, the intestinal handling of glycosylinositol phosphoceramide in mammals remains uncharacterised [134], and whether plant-type long-chain bases are stably detectable in human tissues is unknown [138]. For pharmacological approaches, no agent shown to lower ceramide concentrations in rodents has been evaluated against obesity-related or glycaemic endpoints in humans, and the compounds tested clinically have been developed for oncological, neurological, or lysosomal indications [153,161,163,165].

## 9. Conclusions and Future Perspectives

Sphingolipids, including sphingomyelin in neurons, are bioactive lipids with important physiological functions. This class of lipids is widely involved in various processes such as lipid and glucose metabolism and plays crucial regulatory roles in the development and progression of multiple diseases. Current evidence indicates a close bidirectional relationship between sphingolipid metabolism and obesity. Obesity can disrupt sphingolipid homeostasis through multiple mechanisms, while dysregulated sphingolipid metabolism can further exacerbate obesity and its associated metabolic complications, thereby creating a vicious cycle. Further elucidation of the mechanisms underlying this interaction, and clarification of the central role of sphingolipid metabolism in obesity, may provide novel targets and strategies for the precise prevention and control of obesity and related metabolic diseases. Thus, it has important theoretical significance and clinical value.

However, in this field, progress will depend on moving beyond associative evidence towards tissue-specific, causal mechanisms. This will require species- and compartment-resolved lipidomic analyses of the liver, skeletal muscle, and pancreatic islets, rather than plasma alone [128], together with stable isotope-based approaches capable of distinguishing de novo synthesis, salvage, and turnover pathways [179,180]. The adoption of standardised analytical and reporting methods will also be necessary to enable the comparison of chain-specific ceramides across different studies [181]. The isoform-selective manipulation of the ceramide synthases—currently feasible for CerS1 but not yet for CerS6—would also allow the causal roles of individual ceramide species (with different chain lengths) to be tested directly [14,155].

Such approaches would also facilitate patient selection. Plasma ceramide profiles and composite ceramide scores already predict cardiovascular risk [131,167], but their ability to identify individuals in whom ceramide accumulation contributes to insulin resistance, and who may therefore respond to ceramide-targeted therapies, remains unknown. Species- and ratio-based signatures, such as the C24:0/C16:0 ratio, provide a promising starting point for patient stratification and for enriching future clinical trials [127,168]. The same measurements could also help define the modifiable dietary and microbial inputs to the sphingolipid pathway. Whether these interventions can induce species-specific changes in host tissues, rather than in plasma alone, is a key question that connects the dietary, microbial, and pharmacological aspects of this field.

Ultimately, clinical translation will require human intervention studies that use metabolic endpoints, rather than surrogate endpoints. Isoform-selective ceramide synthase inhibitors and adiponectin receptor agonists that lower ceramide concentrations without changing body weight are rational therapeutic candidates but have not yet entered clinical evaluation for metabolic diseases [155,159]. Likewise, dietary plant glucosylceramide and phytosphingosine warrant controlled clinical studies incorporating measures of adiposity, glycaemic control, and host sphingolipid species [142,145]. At present, sphingolipid-targeted approaches to obesity remain investigational, and identifying which ceramide species, in which tissues, and at which stage of disease are causally involved remains essential for therapeutic translation in the future.

## Figures and Tables

**Table 1:** Principal mechanisms linking sphingolipid metabolism and obesity, alongside the corresponding level of evidence and translational limitations.

Mechanism/Pathway	Evidence Level	Model System	Key Finding	Translational Limitation
Substrate-driven de novo synthesis (SPT)	Human intervention (acute); Rodent; In vitro	Acute lipid infusion in healthy women; rats; HepG2 cells	Fatty acid oversupply increases plasma ceramide and hepatic SPT activity	Acute infusion; not examined under conditions of chronic obesity; small sample
Sphingomyelinase activation (aSMase/nSMase)	Rodent; In vitro	High-fat-fed and ob/ob mice; rats; macrophages and intestinal epithelial cells	Obesity alters sphingomyelinase expression and activity; pharmacological inhibition lowers plasma ceramide levels	Data obtained from rodent tissues; human evidence limited to circulating enzyme activity
Ceramide-mediated inhibition of PKB/Akt	In vitro	Insulin-responsive cell lines; C2C12 myotubes	Ceramide blocks the PH-domain-mediated binding of PKB to 3-phosphoinositides and promotes PP2A-dependent dephosphorylation	Cell culture only; palmitate concentrations often supraphysiological; in vivo relative contribution unclear
ORM/ORMDL-SPT regulation and ERS	Yeast; Rodent (metabolic phenotype only)	*Saccharomyces cerevisiae* ORM mutants; ob/ob mice and mice with diet-induced obesity	Loss of Orm function increases sphingolipid synthesis and activates the UPR; ERS contributes to insulin resistance in mice	Regulatory mechanism demonstrated in yeast; no equivalent rescue experiments in mammalian models
Ceramide-induced apoptosis	In vitro (mainly cancer models)	MCF-7 and glioblastoma cells; MIN6 β-cells	Ceramide channels permit cytochrome c release; chronic palmitate elevations induce ERS and β-cell apoptosis	Data derived from cancer cell lines and biophysical systems; no direct evidence in human islet cells
Mitochondrial injury and fission	Rodent, genetic (causal); Ex vivo organelles; In vitro (cancer models)	CerS6-deficient and CerS2-null mice; isolated rat heart mitochondria; FLT3-ITD AML cells	Ceramide inhibits electron transfer via complex I and reduces complex III activity while increasing reactive oxygen species levels; CerS6-derived C16:0-ceramide binds to mitochondrial fission factor and promotes fragmentation and insulin resistance; ceramide-LC3B-II interaction regulates mitophagy	Fission arm associated with causal effects in mice but remains untested in humans; mitophagy arm derived from cancer models only
Microbial sphingolipid supply (gut microbiota)	Rodent, colonisation and genetic (causal); Human (association; non-randomised intervention)	Gnotobiotic and high-fat-fed mice; human cohort; washed microbiota transplantation	*Bacteroides*- and *Turicibacter*-derived sphingolipids restrain the host ceramide pool; obesity depletes these commensals	Human data associative; microbial sphingolipids not isolated as the mediator; immune effects context-dependent and defined in intestine
Ceramide-enriched membrane platforms	In vitro; Rodent (non-metabolic tissue)	Endothelial cells, macrophages and myotubes; caveolin-enriched microdomains	Platforms co-organise PKB repression, TLR4 assembly and NADPH oxidase assembly	Three arms established separately; not shown co-assembled in obese adipose tissue or liver

Abbreviations: aSMase, acid sphingomyelinase; AML, acute myeloid leukaemia; CerS, ceramide synthase; ERS, endoplasmic reticulum stress; FLT3-ITD, FMS-like tyrosine kinase 3 internal tandem duplication; HepG2, hepatocellular carcinoma cell line G2; LC3B-II, microtubule-associated protein 1 light chain 3 β-II; MCF-7, michigan cancer foundation-7; MIN6 β, MIN6 mouse pancreatic β-cell line; nSMase, neutral sphingomyelinase; ORM/ORMDL, orosomucoid-like protein; PH, pleckstrin homology; PKB/Akt, protein kinase B; PP2A, protein phosphatase 2A; SPT, serine palmitoyltransferase; UPR, unfolded protein response.

**Table 2:** Chain length, enzyme origin, and metabolic context determine the direction and strength of ceramide-induced effects.

Sphingolipid Species (Enzyme)	Principal Metabolic Effect	Direction	Strongest Evidence (Model)
C16:0-ceramide (CerS6)	Insulin resistance in hepatic and adipose tissue, steatosis, and mitochondrial fragmentation	Deleterious	Causal effects in genetic mouse models (CerS6 deletion); correlative data from human cohorts (adipose *CERS6* expression related to body mass index); no human loss-of-function or inhibitor data
C18:0-ceramide (CerS1)	Insulin resistance in skeletal muscle	Deleterious	Causal effects in genetic mouse models (CerS1 deletion); no human data
Very-long-chain C22–C24 (e.g., C24:0; CerS2)	Reducing these species raises C16:0 levels and worsens steatosis and insulin resistance	Neutral or protective	Genetic mouse models (CerS2 haploinsufficiency); human cohort data (C16:0/C24:0 ratio)
C24:1- and C18:1-ceramides	Raising hepatic C24:1 lowers weight and improves glucose tolerance; raising C18:1 attenuates steatohepatitis	Beneficial in these settings	Rodent models
Sphingosine-1-phosphate (S1P)	Counter-regulates ceramide levels via the adiponectin-AdipoR ceramidase axis; the ceramide/S1P balance determines the response	Context-dependent	Rodent gain- and loss-of-function models
Circulating C16:0/C24:0 ratio	Affects cardiac structure, cardiovascular events, and mortality	Biomarker (association)	Human cohort data (FINRISK, PREDIMED, FHS, SHIP)

Abbreviations: AdipoR, adiponectin receptor; CerS, ceramide synthase; FHS, Framingham Heart Study; FINRISK, National FINRISK Study; PREDIMED, Prevención con Dieta Mediterránea; S1P, sphingosine-1-phosphate; SHIP, Study of Health in Pomerania.

**Table 3:** Plant-derived sphingolipids as an exogenous input to host sphingolipid metabolism: reported effects and level of evidence.

Compound	Target Ceramide/Receptor	Metabolic Outcome	Evidence (Model)	Caveat
Plant glucosylceramide (with animal sphingomyelin)	Host ceramide species not examined	Lowers hepatic lipid, non-HDL cholesterol, and insulin levels; glucosylceramide raises adiponectin levels	Rodent (Zucker fatty rats, 45 days)	Genetic obesity model; weight and fat percentage not primary outcome; host ceramide species not examined
Phytosphingosine (oral)	GPR120 agonism (potency similar to α-linolenic acid)	Improves high-fat-diet-induced glucose intolerance	Rodent (mouse)	Not tested against a weight endpoint in obese animals
Phytosphingosine	Host ceramide profile not measured	Lowers total and LDL cholesterol and fasting glucose levels; +9.9% IVGTT K-value; no effect on weight and fat percentage	Human RCT (crossover, 12 men with metabolic syndrome, 4 weeks)	Only human trial with metabolic endpoints; small sample size; circulating and tissue ceramide species were not measured
Plant or koji glucosylceramide	Gut microbiota (*Blautia*); hydrolysed to ceramide	Increases *Blautia* abundance; a glucosylceramidase releases ceramide	In vitro/rodent	Fungal source in one study; no metabolic endpoints; host ceramide profile untested
Plant sphingolipid/phytoceramide (structural)	SPT-ORM1 feedback; anti-inflammatory	Attenuates LPS/TNF-α-induced injury; phytoceramide stabilises ORM1	In vitro; plant/yeast	Almost no mammalian metabolic phenotype data; oral bioavailability not quantified

Abbreviations: GPR120, G protein-coupled receptor 120; IVGTT, intravenous glucose tolerance test; LDL, low-density lipoprotein; LPS, lipopolysaccharide; non-HDL, non-high-density lipoprotein; ORM1, orosomucoid-like protein 1; RCT, randomised controlled trial; SPT, serine palmitoyltransferase; TNF-α, tumour necrosis factor-α.

**Table 4:** Pharmacological and non-pharmacological approaches for modifying sphingolipid metabolism, along with the highest available level of evidence, current clinical status, and principal hindrances to translation.

Target or Intervention	Tool or Exposure	Evidence/Model	Key Metabolic Outcome Reported	Clinical Status
SPT	Myriocin	Rodent	Muscle ceramide lowered and diet-induced insulin resistance reversed in mice	Preclinical only
Ceramide synthases	P053 (CerS1-selective)	Rodent	Muscle fat oxidation increased and adiposity limited; insulin resistance unchanged	Preclinical only
DES1/DEGS1	Des1 antisense knockdown	Rodent	Hepatic insulin sensitivity improved in mice and rats	Preclinical only
Acid sphingomyelinase	Amitriptyline and related agents	Rodent	Liver injury, atherosclerosis, and insulin resistance improved in mice	Approved clinically for depression; no metabolic indication
AdipoR ceramidase	AdipoRon and PEGylated derivatives	Rodent	Tissue and circulating ceramide levels lowered; glucose metabolism improved	Preclinical only
SphK and S1P receptors	Opaganib; fingolimod	Human	Plasma S1P lowered; lipid profile altered in treated patients	Opaganib not approved; fingolimod approved for multiple sclerosis
Glucosylceramide synthase	Genz-123346; eliglustat	Rodent; human	Glucose tolerance, HbA1c levels, and insulin signalling improved in diabetic rodents	Eliglustat approved for Gaucher disease type 1

Abbreviations: AdipoR, adiponectin receptor; CerS, ceramide synthase; DES1/DEGS1, dihydroceramide desaturase 1; HbA1c, glycated haemoglobin; S1P, sphingosine-1-phosphate; SphK, sphingosine kinase; SPT, serine palmitoyltransferase.

**Table 5:** Dietary and lifestyle interventions that modify sphingolipid species, together with the principal reported changes, associated metabolic outcomes, highest available level of evidence and experimental model, and principal caveat.

Intervention	Target Ceramide Species	Metabolic Outcome	Evidence (Model)
Saturated fat	Ceramide raised; C16:0 is most commonly implicated	Insulin resistance; hepatic fat	Human overfeeding; rodent
Mediterranean pattern	Very-long-chain and C18:0-ceramides lowered; C24:0/C16:0-ceramide raised	Endothelial function; CVD risk	Human RCT
Fermentable fibre	Plasma ceramide and hexosylceramide features lowered	Glucose homeostasis	Human trial
*n*-3 PUFA	No species-selective reduction	Lipid mediators; triglycerides	Human RCT
Energy restriction/fasting	C16:0, C18:0, C24:0-ceramide lowered; sphingoid bases lowered	Glycaemic control; adiponectin	Human RCT and cohort
Exercise	Muscle ceramide lowered, independent of weight loss	Insulin sensitivity	Human RCTs

Abbreviations: CVD, cardiovascular disease; PUFA, polyunsaturated fatty acid; RCT, randomised controlled trial.

## Data Availability

No new data were created or analyzed in this study. Data sharing is not applicable to this article.

## Abbreviations

The following abbreviations are used in this manuscript. Abbreviations that have a commonly used descriptive full form are defined at first mention in the text and are listed below. Gene, protein, and cell-line designations that are conventionally used as symbols and have no such descriptive full form (for example, CerS6, CD36, GLUT4, ORMDL, SMPD3, C2C12) are neither expanded in the text nor listed here. All abbreviations and enzyme symbols used in Table 1, Table 2, Table 3, Table 4 and Table 5, including those already defined in the text, are additionally defined in the corresponding table footnotes so that each table can be read independently.

AdipoR1	Adiponectin receptor 1
AdipoR2	Adiponectin receptor 2
aSMase	Acid sphingomyelinase
C1P	Ceramide-1-phosphate
CerS	Ceramide synthase (isoforms CerS1–CerS6)
CoA	Coenzyme A
ER	Endoplasmic reticulum
ERS	Endoplasmic reticulum stress
FFA	Free fatty acid
GIPC	Glycosylinositol phosphoceramide
IL-1β	Interleukin-1β
JNK	c-Jun *N*-terminal kinase
LCB	Long-chain base
LDL	Low-density lipoprotein
LPS	Lipopolysaccharide
NF-κB	Nuclear factor-κB
non-HDL	Non-high-density lipoprotein
nSMase	Neutral sphingomyelinase
nSMase2	Neutral sphingomyelinase 2
PI3K	Phosphoinositide 3-kinase
PKB	Protein kinase B
PP2A	Protein phosphatase 2A
S1P	Sphingosine-1-phosphate
SphK	Sphingosine kinase (isoforms SphK1, SphK2)
SPT	Serine palmitoyltransferase
TLR4	Toll-like receptor 4
TNF-α	Tumour necrosis factor-α
UPR	Unfolded protein response
